# Mining Structural Information from Gas Chromatography-Electron-Impact Ionization-Mass Spectrometry Data for Analytical-Descriptor-Based Quantitative Structure–Activity Relationship

**DOI:** 10.3390/jox15060177

**Published:** 2025-11-01

**Authors:** Yasuyuki Zushi

**Affiliations:** 1 Research Institute of Science for Safety and Sustainability, National Institute of Advanced Industrial Science and Technology, 16-1 Onogawa, Tsukuba 305-8569, Japan; zushi.yasuyuki@aist.go.jp; Tel.: +81-50-3522-6171; 2 Graduate School of Science and Technology, University of Tsukuba, 1-1-1 Tennodai, Tsukuba 305-8577, Japan

**Keywords:** quantitative structure–activity relationship (QSAR), mass spectra, gas chromatography-electron-impact ionization-mass spectrometry (GC-EI-MS), t-distributed stochastic neighbor embedding (t-SNE), analytical-descriptor-based model, unknown chemicals

## Abstract

Recently developed quantitative structure–activity relationship (QSAR) prediction uses machine learning techniques with analytical signals from the full scan of mass spectra as input, and does not need exhaustive structural determination to assess unknown compounds. The QSAR approach assumes that a mass spectral pattern reflects the structure of a target chemical. However, the relationship between the spectrum and structure is complex, and requirement of its interpretation could restrict further development of QSAR prediction methods based on analytical signals. In this study, whether gas chromatography-electron-impact ionization-mass spectrometry (GC-EI-MS) data contain meaningful structural information that assists QSAR prediction was determined by comparing it with the traditional molecular descriptor used in QSAR prediction. Four molecular descriptors were used: ECFP6, topological descriptor in CDK, MACCS key, and PubChem fingerprint. The predictive performance of QSAR based on analytical and molecular descriptors was evaluated in terms of molecular weight, log *K_o-w_*, boiling point, melting point, water solubility, and two oral toxicities in rats and mice. The influential variables were further investigated by comparing analytical-descriptor-based and linear regression models using simple indicators of the mass spectrum. The investigation indicated that the analytical and molecular descriptors preserved structural information differently. However, their performance was comparable. The analytical-descriptor-based approach predicted the physicochemical properties and toxicities of structurally unknown chemicals, which was beyond the scope of the molecular-descriptor-based approach. The QSAR approach based on analytical signals is valuable for evaluating unknown chemicals in many scenarios.

## 1. Introduction

A large number of chemical structures can be synthesized practically, as evidenced by the Chemical Abstracts Service (CAS) registering over 200 million synthesized chemicals since the 1960s [1]; moreover, the Chemical Space Project has generated more than 166 billion in silico chemicals [2]. Thousands of chemical signals can be detected in a single analysis by gas chromatography combined with mass spectrometry (GC-MS) using the recently developed non-target screening methods for chemicals present in various environmental media such as river water, sediment, and the atmosphere [3,4,5,6]. Unfortunately, the structures of most chemicals detected by such non-target analyses using high-mass-resolution high-scan-rate mass spectrometers remain unknown. Recently, a new approach to quantitative structure–activity relationship (QSAR, also known as QSPR prediction) which uses physicochemical properties as the subject, has been proposed. The approach utilizes machine learning (ML) techniques with analytical signals—the full scan of mass spectra—as input. This approach has been applied to gas chromatography combined with electron-impact ionization mass spectrometry (GC-EI-MS) by using the XGBoost linear algorithm as the ML algorithm. It has also been applied to liquid chromatography combined with high-resolution tandem mass spectrometry by using the xgbDART algorithm as the ML algorithm with combined molecular descriptors derived from analytical signals processed using the SIRIUS+CSI:FingerID software [7,8,9]. One approach involving the use of both the retention time index (RI) and mass spectrum of GC-EI-MS is the analytical-descriptor-based QSAR method, which is termed Detective-QSAR [9]. This approach has enormous potential for application in environmental chemical risk analysis because it can be used to assess numerous detected unknowns by mass-spectrometry-based non-target analysis without exhaustive structure determination, which is essential for the traditional (i.e., molecular-descriptor-based) QSAR prediction. Moreover, the approach can be utilized for environmental analysis and in fields such as drug discovery, epidemiology, functional material production, and food and beverage safety control. In this approach, a mass spectral pattern is assumed to reflect the structure of a target chemical. This assumption is not immediately contradictory based on intuitive perception and the resulting high predictive performance of the proposed approach. However, the approach implemented as Detective-QSAR [10], which is a direct prediction model that uses RI and the GC-EI-MS profile as inputs, employs an ML technique that generates a complex model structure. Interpreting such ML-based QSAR models is challenging because it requires identifying which input variables (i.e., substructures) influence the results. In addition, other concerns abound, especially when utilizing low-mass-resolution GC-EI-MS. For example, assigning the correct substructure to each mass spectral bin is sometimes difficult, and the hard ionization of EI generates many fragment ions from the original molecule. This complexity makes it difficult to establish the model structure that follows the QSAR concept of the similarity–property principle [11], thereby limiting the in-depth exploration of the approach.

Given this context, this study sought to examine whether GC-EI-MS data contain structural information that simplifies QSAR prediction. As mentioned above, electron impacts cause hard ionization and do not always generate a spectrum of substructure that easily ensures direct correlation with the molecular ion. Therefore, finding clear one-to-one relationships linking generated GC-EI-MS fragments and substructures is generally difficult. To explore whether the structural information that is effective for prediction is preserved, chemical groups are first assigned to each chemical linked to the GC-EI-MS data using ClassyFire, which can define the chemical group of a candidate based on structural information obtained from the simplified molecular input line entry system (SMILES) notation and CAS registry number [12]. This approach enables confirmation of whether the same chemical group reflects EI mass spectra with a similar pattern. The performance of the analytical-descriptor-based QSAR model is then evaluated by comparing it with that of the six molecular-descriptor-based QSAR model. The GC-EI-MS data used for modeling are also investigated to understand their influence on the prediction in Detective-QSAR by comparing the four different linear regression models. Based on the results of these analyses, the relevance of the analytical-descriptor-based QSAR approach for chemical assessments was successfully determined.

## 2. Materials and Methods

### 2.1. Dataset and Preparation

Data on the chemicals analyzed in the GC-EI-MS scan mode were obtained from NIST17, MassBank, Fiehn laboratory, RIKEN, and experimental measurement data for system evaluation [9,13,14,15,16,17]. Chemicals with mass spectral, RI, and SMILES information were extracted as a data list and then curated into a chemical list based on physicochemical properties or toxicities for further analysis. Here, the RIs obtained from a semi-polar column, which is typically specified as the (5%-phenyl)-methylpolysiloxane stationary phase, under ramped temperature conditions were subject to selective inclusion; when this information was unavailable, that of a non-polar column was selected as an alternative candidate. The mass spectral scan range was individually configured according to the fragment generation range of each compound, resulting in a compound-specific value (See Supplementary Materials for the range of each compound). The properties of log *K_o-w_*, boiling point (BP), melting point (MP), water solubility (WS), and toxicities of the median lethal dose (LD_50_) administered orally to rats and mice were obtained from ChemID*plus* and Comptox [18,19]. The molecular weights (M_w_) of the chemicals were obtained using OPERA [20]. The M_w_, WS, and LD_50_ values, after conversion into logarithmic scales, were denoted as log M_w_, log WS, and log LD_50_, respectively. The total numbers of lists were 12810, 3385, 3836, 2674, 1299, 1383, 2080, and 1630 for M_w_, BP, MP, log *K_o-w_*, WS, LD_50_ (rat, oral), and LD_50_ (mouse, oral), respectively. In the present study the values of the vapor pressure were not presented. Detective-QSAR was developed based on the dataset, and the hyperparameter optimization, train/validation/test data splitting, and calculation of applicability domain are detailed elsewhere [9]. To provide an overview of data coverage in Detective-QSAR, chemical lists with properties used for the development were included in Supplementary Data (See Supplementary Materials). The molecular-descriptor-based QSAR was developed in a consistent manner to enable comparison. Various molecular descriptors—ECFP6 (called circular in some cases), MACCS key, PubChem fingerprint, and topological descriptor in CDK, which were used as input variables for QSAR, were calculated based on SMILES using the R package rcdk (version 3.7.0) [21,22]. The term “molecular descriptor” includes the more specific categories of structure key and hashed fingerprint as well as descriptors of general type. Hashed fingerprints can be further divided into path-based fingerprints and circular fingerprints, depending on the algorithm for the vector generation [23]. Both the structure key and hashed fingerprint, which are represented by bit elements, constitute “fingerprints” in a broad sense. The structure key requires a substructure library to generate the vector representing the molecular structure of interest. By contrast, a hand-hashed fingerprint can generate the vector without the library but only by using a certain computer algorithm. ECFP6 is categorized as a hashed fingerprint and is based on the Morgan algorithm for fingerprint generation. The hashed fingerprint can be applied to a wide range of chemicals because no prior substructure candidates are required. The MACCS key, which is a structure key, is a well-known molecular descriptor with a 166-bit vector constructed from a substructure library. The PubChem fingerprint (which is actually a structure key) was developed for structure searching using the large database of PubChem [24]. The PubChem fingerprint is effective in QSAR prediction as well. Unlike the other molecular descriptors listed above, the topological descriptor in CDK is neither a structure key nor a hashed fingerprint but is a general descriptor, which comprises continuous variances in the substructures of the molecule and physicochemical properties calculated based on structural features. Further details of the molecular descriptors have been provided elsewhere [23].

The chemicals were classified using the web version of ClassyFire [25], which categorizes chemicals into particular classes depending on their structure. The categories were constructed by referring to the biological classification scheme: kingdom, superclass, class, and subclass. The chemicals used in this study were classified into approximately 20 classes, depending on the dataset of interest. The dataset were aligned with Detective-QSAR, but items that were not categorized by ClassyFire were excluded, resulting in 0–4% fewer entries.

### 2.2. Data Analysis

Data clustering was performed for the chemicals using t-distributed stochastic neighbor embedding (t-SNE) in the R package t-SNE (version 0.1-3.1) [26,27]. t-SNE is used for the dimensional compression of high-dimensional data. t-SNE determines the position of each plot in a lower-dimensional (e.g., 2D or 3D) map based on variable similarity among the data. The position is calculated to ensure similar data are placed in nearby regions according to an iterative calculation with loss minimizing. Therefore, the scale of the map is distorted to emphasize the relative closeness of the points.

Briefly, the process of dimensional compression minimizes the loss function:

(1) $$L=\sum_{i}\sum_{j}p_{ij}\log\frac{p_{ij}}{q_{ij}},$$

where $p_{ij}$ represents pairwise affinity of points $x_{i}$ and $x_{j}$ in high-dimensional space, and $q_{ij}$ represents that of points $y_{i}$ and $y_{j}$ in low-dimensional space. The affinities $p_{ij}$ and $q_{ij}$ are calculated from

(2) $$p_{ij}=\frac{p_{i|j}+p_{j|i}}{2n},$$

(3) $$q_{ij}=\frac{{\left(1+{\left\|y_{i}-y_{j}\right\|}^{2}\right)}^{-1}}{{\sum_{k\neq i}\left(1+{\left\|y_{i}-y_{j}\right\|}^{2}\right)}^{-1}},$$

where

(4) $$p_{j|i}=\frac{exp\left(-{\left\|x_{i}-x_{j}\right\|}^{2}/{2\sigma }_{i}^{2}\right)}{\sum_{k\neq i}exp\left(-{\left\|x_{i}-x_{k}\right\|}^{2}/{2\sigma }_{i}^{2}\right)}.$$

The most desirable case is when *L* = 0, i.e., when the distributions of *p* and *q* are the same. The position of *y* at iteration *t* is updated according to the stochastic gradient descent with learning rate *η* and scaling factor α as follows:

(5) $$y^{\left(t\right)}=y^{\left(t-1\right)}+\eta \frac{\partial L}{\partial y}+\alpha \left(t\right)\left(y^{\left(t-1\right)}-y^{\left(t-2\right)}\right).$$

The hyperparameter of the initial dimension of the dataset and perplexity, which represents the optimal number of neighbors in the t-SNE map, were both set to the default value of 30 after grid search for preliminary inspection.

For comparison, two different types of inputs were prepared for clustering by t-SNE: an analytical descriptor (GC-EI-MS data of RI with mass spectrum) and molecular descriptors (ECFP6, MACCS key, PubChem fingerprint, or topological descriptor in CDK). Each *m*/*z* intensity and RI was standardized with the mean and standard deviation and used as a standardized analytical descriptor prior to the t-SNE calculation. The topological descriptor was also standardized because it consists of continuous variables (unlike the other molecular descriptors with bit representations). Datasets of analytical and molecular descriptors were also used to build a QSAR prediction model based on XGBoost [28]. Prediction models using each molecular descriptor as input were prepared in this study and compared to Detective-QSAR, that uses the analytical descriptor as input [9]. The analytical descriptor was not standardized for construction of Detective-QSAR, unlike that for t-SNE, because standardization does not affect the calculation results obtained by XGBoost, unlike those acquired by t-SNE. Furthermore, the topological descriptor, which consists of continuous values, was not standardized for the XGBoost model. The performances of the analytical- and molecular-descriptor-based models were compared. Another analytical-descriptor-based model that uses GC-EI-MS data without RI (Detective-QSAR without RI) was prepared. In total, six models (four ML models using various molecular descriptors, Detective-QSAR, and Detective-QSAR without RI) were prepared for performance evaluation in terms of six properties and two toxicities. Linear regression models that use the indicators of GC-EI-MS data as explanatory variable(s) were also prepared for comparison with Detective-QSAR. Statistical models, including linear regression models, are well-suited for hypothesis testing and causal interpretation, offering explicit model structures and interpretability. Therefore, comparative analysis with statistical models was performed to elucidate the architecture of the machine learning models. The center of *m*/*z* value in the mass spectrum (center_mz), *m*/*z* of the highest intensity (maxint_mz), highest *m*/*z* (max_mz), standard deviation of *m*/*z* (sd_mz), number of spectral bins (bin_num), RI, or a combination of these variables were used as representatives of the GC-EI-MS data.

The training dataset was consistent among all models with the same subject, except for a few cases of data removal because of incomplete data. Model performance was evaluated using a combination of the validation and test datasets because the validation dataset did not have a marked influence on model performance in the previous study [9].

## 3. Results and Discussion

### 3.1. Relationship Between Chemical Class and Constructed Cluster

**Figure 1:**
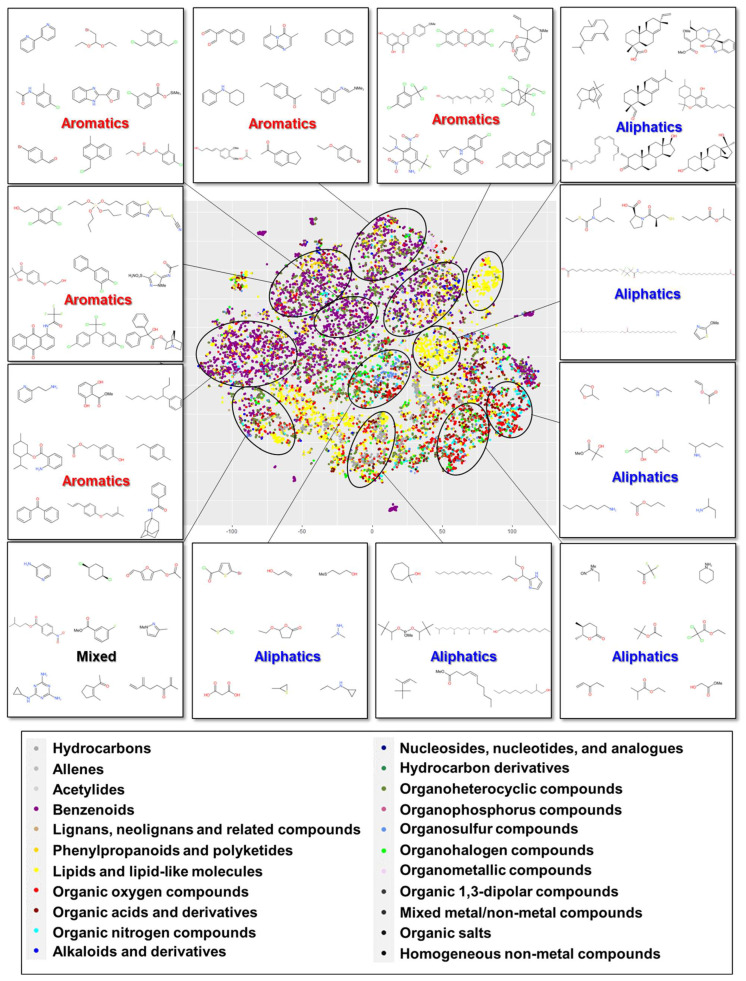
t-SNE map based on standardized analytical descriptor. The plot illustrates GC-EI-MS data with RI that are available for the QSAR modeling (n = 12,859). The chemical structures of randomly selected plots from corresponding clusters are depicted in the small windows.

The hypothesis that GC-EI-MS data contain meaningful structural information was explored by clustering datasets of standardized analytical descriptors (RI with mass spectrum) and molecular descriptors that were linked to chemical classes using ClassyFire. Figure 1 shows the results of clustering by t-SNE on the standardized analytical descriptor.

Five of the 12 circled clusters in the map consisted mainly of aromatics, and six clusters consisted of aliphatics; one cluster was a mix of aromatics and aliphatics. In terms of superclass, five clusters that consisted mainly of benzenoids were placed in the upper left of the map. Lipids and lipid-like molecules formed several clusters from the upper right to the bottom left. Organic oxygen and nitrogen compounds were concentrated at the bottom right. The positions of the chemicals in each superclass are shown in Figure S1 in the Supplementary Material.

By contrast, the t-SNE map based on the analytical descriptor without standardization exhibited small clusters around the edge (Figures S2 and S3). Standardization prior to t-SNE analysis assists in capturing the deviation in each variant compared with the use of raw input, reducing localization in small clusters. The results with the standardized analytical descriptor more closely resembled those with the molecular descriptor (PubChem fingerprint) shown in Figure S4. As in the t-SNE map obtained using the standardized analytical descriptor, 5 of the 12 circled clusters in the t-SNE map based on the PubChem fingerprint consisted mainly of aromatics, and the others comprised aliphatics. In terms of superclass, three clusters that consisted mainly of benzenoids were located on the left in the map. Lipids and lipid-like molecules formed several clusters on the right. Organic oxygen compounds were widely distributed in the map; organic nitrogen compounds were concentrated in the center to the right (Figure S5). Compared with that of the map from the analytical descriptor, this map exhibited more separation according to superclasses such as organosulfur compounds, organoheterocyclic compounds, hydrocarbons, phenylpropanoids and polyketides, and organic nitrogen compounds; therefore, chemicals from multiple chemical classes did not frequently overlap. Similar results were obtained with other molecular descriptors (ECFP6, MACCS key, and topological descriptor). Clustering was performed with over 49,000 samples based on the standardized analytical (mass spectrum without RI to increase the number of samples) and molecular descriptors (PubChem fingerprint) to ensure consistency with the increased sample number. A similar trend was observed in the cluster formation with a large number of samples (Figures S6 and S7).

A comparison of the results obtained using the analytical and molecular descriptors showed that both types of descriptors substantially preserved the structural features of chemicals but differed slightly in the manner in which it was performed. The molecular descriptors appeared to differentiate chemicals more sensitively by chemical functional group and certain substructures, as clearer cluster separations were observed according to superclass (Figure S7). The standardized analytical descriptor tended to mix different classes of chemicals as long as the spectral information of the chemicals were similar (Figure S6). This difference between the descriptor types could generate a gap in the predictive performance of QSAR.

The spectral similarity between and within clusters was investigated to capture the characteristics of clustering trends for analytical and molecular descriptors. Table S1 lists the similarities in the standardized mass spectrum calculated using the cosine distance on each cluster (see Figure S8 for the respective cluster IDs). The analytical descriptor exhibited relatively high similarity with respect to the standardized mass spectrum among cluster members (Clusters 3a–8a; 0.14–0.32), except for the cluster in which aromatics predominated or a mix existed (Clusters 1a, 2a, 9a–12a; 0.03–0.12). Conversely, the inter-cluster similarities were substantially lower (Clusters 1a–12a; (−0.08)–0.16).

The molecular descriptor (PubChem fingerprint) displayed relatively high similarity with respect to the standardized mass spectrum within cluster members (Clusters 1b–7b; 0.15–0.43), except for the clusters in which aromatics predominated (Clusters 8b–12b; 0.06–0.11). In contrast to the case using analytical descriptors, similarity between clusters in close proximity, i.e., neighboring clusters, remained relatively high for aliphatics (0.14–0.34), as shown in Table S2 (see Figure S9 for the respective cluster IDs).

Although clustering based on both the molecular and analytical descriptors successfully grouped together chemicals with similar mass spectral patterns, clustering based on analytical descriptors enabled better discrimination (low similarity) among chemicals. Moreover, high similarity was not observed within the same chemical class (0.009–0.17) (Table S3). Therefore, as indicated by the results of analytical-descriptor-based clustering, chemicals of the same class do not always have a similar spectrum and do not aggregate on the t-SNE map.

**Figure 2:**
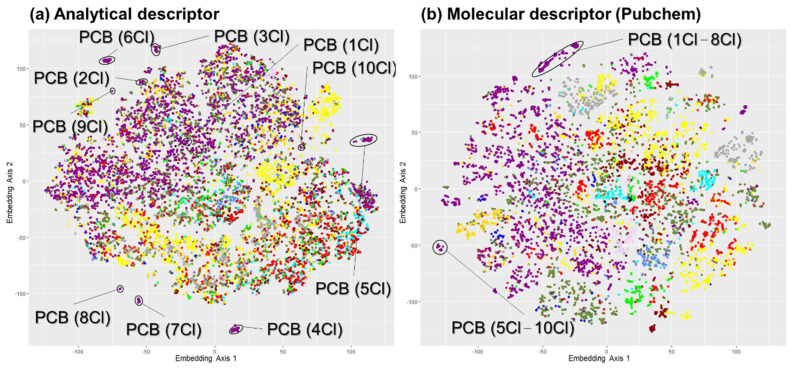
Difference in distribution of PCBs given by standardized analytical and molecular descriptors. The plots consist of 12,859 data for (**a**) analytical descriptor, and 12,785 data for (**b**) molecular descriptor.

Another difference between analytical-descriptor-based and molecular-descriptor-based clustering is the effect of a slightly different structure. A slight variation in structure, such as an increase in the number of substituents, induces a spectral shift that causes the assigned clusters to be different in analytical-descriptor-based clustering. Figure 2 shows the differences in the cluster formation of polychlorinated biphenyls (PCBs) with various numbers of chlorine atoms between the analytical and molecular descriptors.

The clusters of PCBs with 1–8 and 5–10 chlorine atoms were aggregated in each cluster when using molecular-descriptor-based clustering (PubChem fingerprint), whereas different clusters formed in analytical-descriptor-based clustering because of similar but shifted mass spectral patterns. The same trend was observed for other molecular descriptors (ECFP6, MACCS key, and topological descriptor). The result indicates that converting the analytical descriptor to a vector (which mitigates the effect of the extent of the *m*/*z* shift and emphasizes locally analogous spectral patterns that are positionally shifted, such as dimension-invariant vector or estimated latent variables) would refine its capability to represent molecular features that potentially lead to higher performance in property estimation. These differences between analytical and molecular descriptors were observed to gain an overview of the t-SNE maps. The Silhouette index, Adjusted Rand Index, or other types of indexes would more effectively enable precise investigation and comparisons for fair differences and will be utilized for further detailed surveys.

### 3.2. Model Performance Using Analytical and Molecular Descriptors

The performance of the QSAR models using analytical and molecular descriptors was evaluated on various physicochemical properties and toxicities. The ML models corresponding to the target property or toxicity were trained using each descriptor. The performance of Detective-QSAR was comparable to or better than those of the models based on molecular descriptors for BP and MP in terms of the root-mean-square error (RMSE) (Table 1). The mean absolute error (MAE) is presented in Table S4, and the amount of training data is provided in Table S5. The performance in terms of log *K_o-w_* and log WS was better for the model based on the molecular descriptor. The log LD_50_ performance for rats and mice was marginally better for the molecular-descriptor-based model, whereas the log M_w_ performance achieved by most molecular-descriptor-based models was comparable to that obtained by the analytical-descriptor-based model. The direct prediction of LD_50_ based on GC-EI-MS is supported by the preservation of the structural information in the RI and mass spectrum, which aligns with the principles of structure–activity relationships. From the perspective of data quality, ChemID*plus* [19], which served as the information source of LD_50_ in this study, rigorously managed reliable toxicological data; therefore, confounding factors, including species differences, are considered to be systematically controlled to ensure data integrity. However, given the experimental context, variability in toxicological data, particularly in LD_50_, is not unusual; therefore, a certain extent of discrepancies in the model prediction must be acknowledged. To enhance the prediction performance on the toxicity across a wider range, increasing the amount of training data would be important. Adopting an alternative perspective, exploring predictive models on NOALE for model species with simple biology or in vitro assays would be worth exploring.

The performance with the topological descriptor (0.005) exceeded those of the other analytical and molecular descriptors (0.04–0.06) by an order of magnitude because topology encompasses variables related to molecular size/weight that have been calculated from the chemical structure that was used as input [29] (i.e., the prediction of log M_w_ using the molecular descriptor was verified only to clarify the model mechanisms because M_w_ is evidently obtained with the molecular structure as input for the molecular-descriptor-based model). The reason for the comparable or better performance of the analytical-descriptor-based model in terms of BP and MP is that the measured RIs contained in this descriptor are thermodynamically related to these properties. The inferior performance in terms of log *K_o-w_* and log WS, which reflect interaction with the water phase, may have occurred because Detective-QSAR was trained on the RIs of non-polar or semi-polar GC columns. These columns mainly capture chemical interactions related to hydrophobic interaction. Consequently, they have difficulty in capturing other interactions of hydrogen and ionic bonds that are likely to generate in the water phase [9,30], resulting in inferior performance unlike those of BP and MP.

The model with the combined use of molecular descriptors can outperform the analytical-descriptor-based model, as reported in previous studies [7,31]; their individual use yields a performance comparable to that of the analytical-descriptor-based model in terms of log M_w_ (except with the topological descriptor), BP and MP.

**Figure 3:**
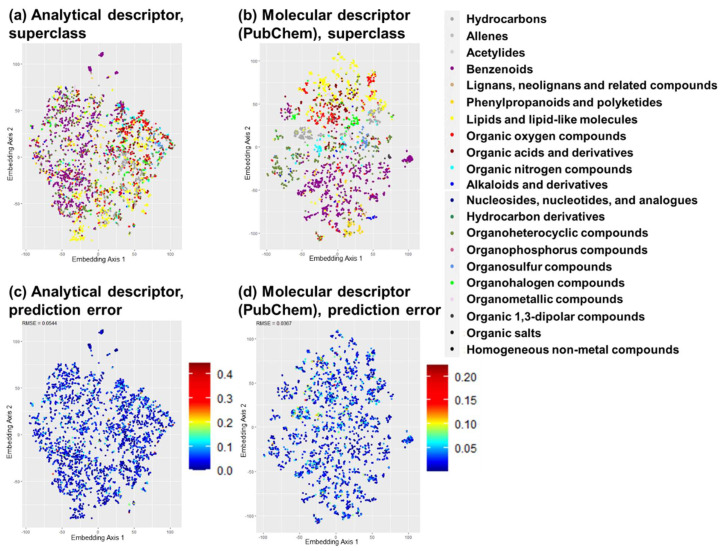
Distribution of prediction error of QSAR models on log M_w_. (**a**) t-SNE map based on the standardized analytical descriptor with log M_w_ test data, (**b**) t-SNE map based on a molecular descriptor (PubChem fingerprint) with log M_w_ test data, (**c**) t-SNE map of (**a**) layered with the absolute error of the Detective-QSAR model prediction of log M_w_, (**d**) t-SNE map from (**b**) layered with the absolute error on the molecular-descriptor-based QSAR model prediction (PubChem fingerprint) on log M_w_.

Absolute errors in prediction were plotted on a t-SNE map to investigate whether these models had weaknesses in specific classes/clusters (Figure 3). No bias or class/cluster with poor prediction was found upon comparing the original t-SNE map with the class and the map with the absolute error. Consistent results were observed for the other properties and molecular descriptors; therefore, all models performed well in predicting properties and toxicities, regardless of the chemical class.

Figure 2 shows that homologous chemicals were recognized differently by the analytical and molecular descriptors. The shift in the mass spectrum by a slight transformation of the structure, such as substitution, can influence the prediction performance and/or coverage of chemical space (i.e., the AD of the model) in the analytical-descriptor-based model. This effect occurs because these homologous chemicals are treated as heterogeneous data during training by the XGBoost model and individually contribute to model building without interactively reinforcing the model. Certain data conversions (e.g., word2vec-based data embedding [32]) will enable their recognition as homologues. Such conversions can further enhance the precision and AD of analytical-descriptor-based models. Therefore, these points should be investigated further in future studies.

### 3.3. Influential Features for Prediction Based on Analytical Descriptor

Understanding the prediction mechanism of the analytical-descriptor-based model fully is a challenge as it was only recently developed. Linear regression models with the explanatory variables center_mz, maxint_mz, max_mz, sd_mz, bin_num, RI, or a combination were compared in terms of RMSE; the models displaying higher performances are listed in Table 2. The corresponding MAE values are listed in Table S6, and the amount of training data is provided in Table S7.

Each model using a particular property had a dominant variable when compared with the model that used all variables. For example, the RMSE of the linear regression model in terms of log M_w_ was comparable to those of the model with a single maxint_mz variable (0.064) and the all-six-variable model (0.062), which indicates the importance of a symbolic *m*/*z* signal in the mass spectrum. Similarly, for log *K_o-w_*, maxint_mz (1.55) was comparable to that in the all-six-variable model (1.48).

For BP, the RI (29.3) was comparable to that of the all-six-variable model (29.1), which indicates the importance of structural molecular interactions related to molecular shape. In terms of log WS, center_mz (1.76) was comparable to that of the all-six-variable model (1.68), which indicates the importance of balancing the entire mass spectral pattern. Among the toxicity models subtle differences were observed, e.g., for log LD_50_ (rat, oral), maxint_mz (0.79) was similar to the value obtained with the all-six-variable model (0.79). A dominant feature in GC-EI-MS data was found on each model for each property; the type of feature depended on the target property. Conversely, when full GC-EI-MS information was used as applied in Detective-QSAR, it overwhelmed all linear regression models with dominant variables or all six variables for all target properties as shown in Table 2. The predictive performance of the all-six-variable model in terms of log M_w_ and BP was inferior to the linear regression model when the RI was excluded from Detective-QSAR. RI represents a feature of the chemical structure and contributes to the predictive performance of these analytical-descriptor-based models. The investigations in this study suggest that GC-EI-MS data contain features representative of the chemical structure.

## 4. Conclusions

This study identified the differences and similarities of information regarding the chemical structure of compounds obtained from analytical and molecular descriptors using clustering by t-SNE. Both descriptors were largely able to separate aromatics and aliphatics. The molecular descriptor was more efficacious in formulating clusters according to the chemical superclass in the t-SNE clustering. Although differences existed between the descriptors in the manner in which information was preserved, both include important features that contribute to higher precision in predicting physicochemical properties and toxicities. The analytical-descriptor-based model performed better when evaluating materials with unknown structures; the molecular-descriptor-based models performed slightly better in terms of properties such as log *K_o-w_*, log WS, and log LD_50_. The analytical-descriptor approach is currently limited by the direct signals of fragment ions with unit mass; this limitation may restrict a performance upgrade of the QSAR model and t-SNE. This issue may be overcome by using specific data conversions or exploring latent variables and word2vec embeddings or other transformer models that use the encoder–decoder structure; moreover, the AD of the prediction model could be expanded. Further studies on analytical descriptors will expand the application scope of the models to a wider range of scenarios, such as environmental monitoring, toxicological risk assessment, drug discovery, epidemiological surveys, material development, and food and beverage safety control, in which chemicals must be detected and evaluated even in the absence of structural information.

## Figures and Tables

**Table 1:** RMSE-based predictive performance of QSAR models based on analytical (Detective-QSAR) and molecular descriptors.

	Detective-QSAR	Topological Descriptor	ECFP6	MACCS Key	PubChem Fingerprint
log M_w_	0.041	0.005	0.052	0.060	0.037
log *K_o-w_*	1.02	0.53	0.81	0.76	0.58
BP	23.5	21.2	42.9	39.0	26.8
MP	52.1	42.0	53.1	50.4	44.2
log WS	1.40	0.82	1.22	1.10	0.85
log LD_50_ (rat, oral)	0.72	0.61	0.65	0.60	0.56
log LD_50_ (mouse, oral)	0.67	0.55	0.58	0.55	0.55

M_w_: molecular weight; *K_o-w_*: partitioning coefficient between octanol and water; BP: boiling point; MP: melting point; WS: water solubility; LD_50_: median lethal dose.

**Table 2:** RMSE-based predictive performance on GC-EI-MS indicators for analytical-descriptor-based QSAR model and regression models.

	Detective-QSAR	Detective-QSAR (Without RI)	All-Six-Variable Model	maxint_mz	RI	center_mz
log M_w_	0.041	0.132	0.062	0.064	0.099	0.134
log *K_o-w_*	1.02	0.95	1.48	1.55	1.75	1.59
BP	23.5	40.7	29.1	61.8	29.3	67.7
MP	52.1	56.9	61.3	79.6	65.6	76.5
log WS	1.40	1.26	1.68	1.84	2.03	1.76
log LD_50_ (rat, oral)	0.72	0.67	0.79	0.79	0.80	0.82
log LD_50_ (mouse, oral)	0.67	0.64	0.68	0.68	0.70	0.70

All-six-variable model: center_mz + maxint_mz + max_mz + sd_mz + bin_num + RI. center_mz: center of *m*/*z* value in the mass spectrum, maxint_mz: *m*/*z* of the highest intensity, max_mz: highest *m*/*z*, sd_mz: standard deviation of *m*/*z*, bin_num: number of spectral bins, RI: retention index.

## Data Availability

The codes for QSAR modeling and t-SNE mapping on molecular descriptors and the list of chemicals with chemical classes and SMILES for clustering (SMILES_list_for_Clustering.xlsx) are available on GitHub at https://github.com/Yasuyuki-Zushi/Mining-Structural-Information (accessed on 13 October 2025). The label data for the QSAR models, including BP, MP, log *K_o-w_*, WS, LD_50_ (rat, oral), and LD_50_ (mouse, oral), were obtained from ChemID*plus* (recently translocated to the database of PubChem) and Comptox. The data for training, validating, and testing the analytical-descriptor-based QSAR model included the NIST17 commercial library.

## Supplementary Materials

The following supporting information can be downloaded at https://www.mdpi.com/article/10.3390/jox15060177/s1; Figures S1–S9 and Tables S1–S7 are available as a word file, and index of training and validation data to exhibit data range in Detective-QSAR are available as csv files.

## Abbreviations

The following abbreviations are used in this manuscript:

AD	Applicability domain
BP	Boiling point
CAS	Chemical Abstracts Service
*K_o-w_*	Octanol-water partitioning coefficient
MAE	Mean absolute error
ML	Machine learning
MP	Melting point
M_w_	Molecular weight
RI	Retention time index
RMSE	Root-mean-square error
SMILES	Simplified molecular input line entry system
WS	Water solubility
