# Supplementary material for: Mining Structural Information from Gas Chromatography-Electron-Impact Ionization-Mass Spectrometry Data for Analytical-Descriptor-Based Quantitative Structure–Activity Relationship
Source: J Xenobiot. 2025 Nov 1;15(6):177. doi: 10.3390/jox15060177 (PMC12641815; doi:10.3390/jox15060177)
Supplement: Supplementary file 1 [file jox-15-00177-s001.zip › jox-3866436-Supplementary_Material.pdf]

Supplementary Material

Mining structural information from gas chromatography-electron-impact ionization-mass spectrometry data for analytical-descriptor-based quantitative structure–activity relationship

Yasuyuki Zushi [1,2*]

[1] *Research Institute of Science for Safety and Sustainability, National Institute of Advanced Industrial Science and Technology, Japan*

[2] *Graduate School of Science and Technology, University of Tsukuba, Japan*

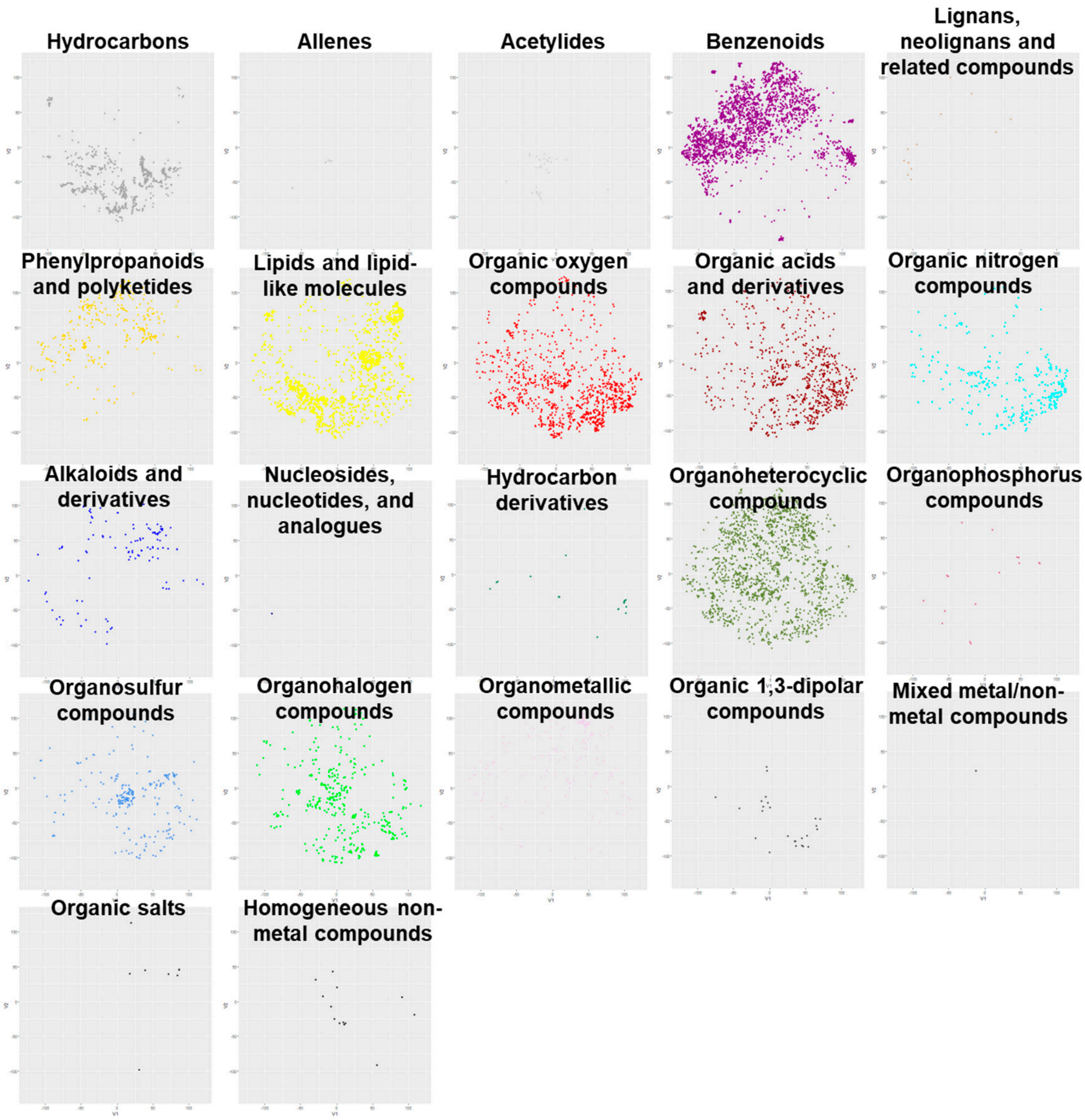

Figure S1. t-SNE map based on the standardized analytical descriptor for each superclass using ClassyFire.

The colors of the plots indicate the superclasses of chemicals obtained using ClassyFire. The circled clusters were designated as aliphatics, aromatics, or mixed, based on the chemical structures of their predominant members. See Figure 1 for color legends.

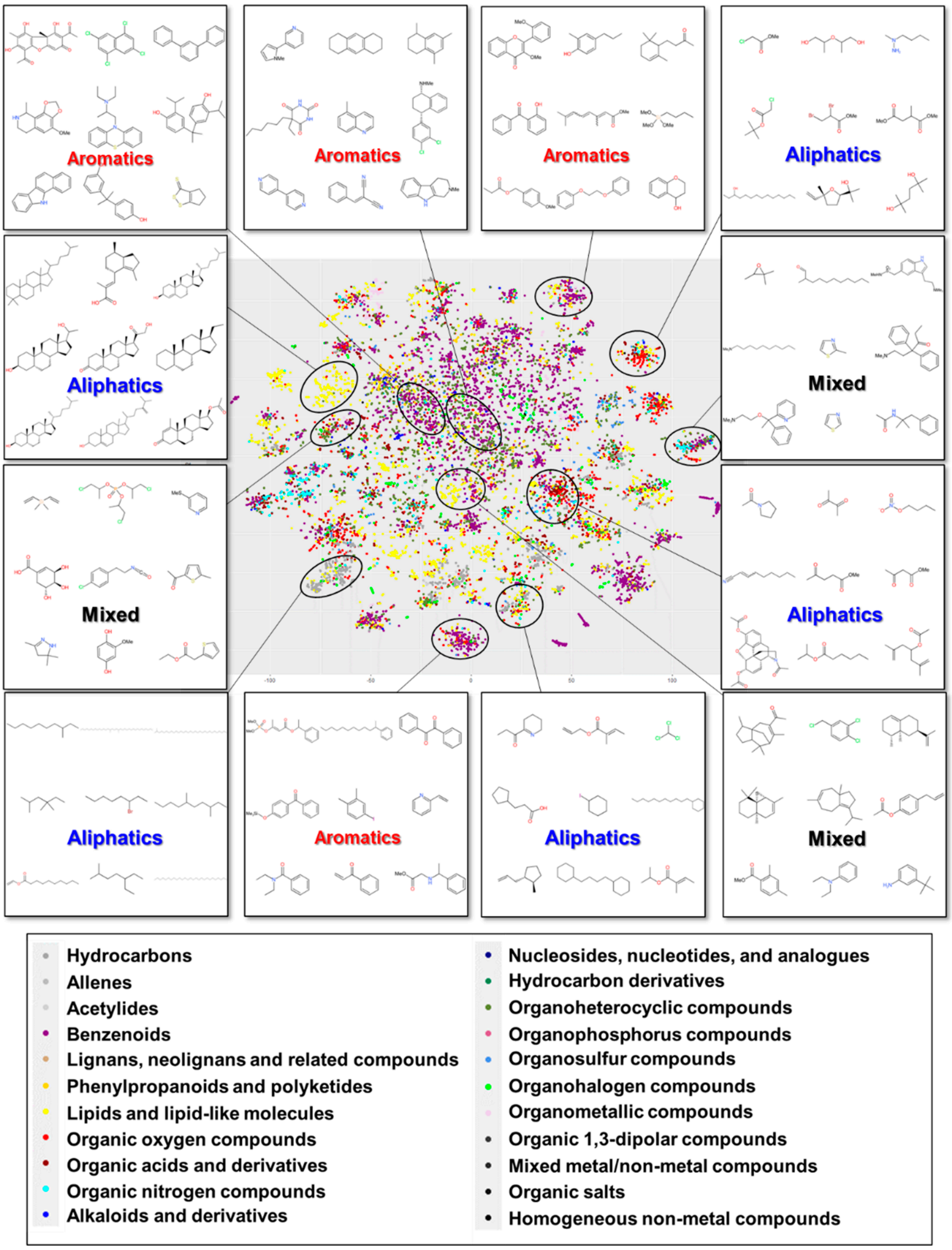

Figure S2. t-SNE map based on the analytical descriptor without standardization

The plot consists of GC-EI-MS data with RI that are available for the QSAR modeling (n = 12859).

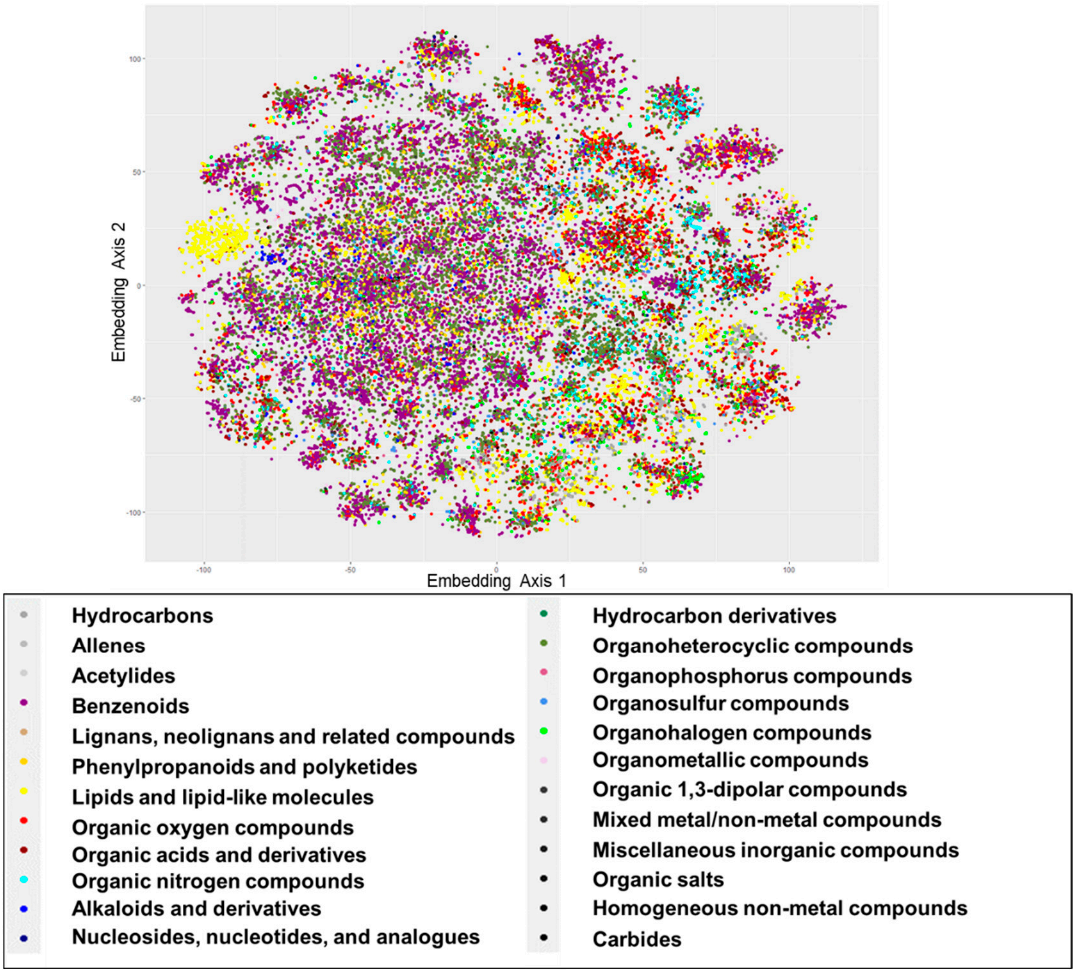

Figure S3. t-SNE map based on the analytical descriptor without standardization

The plot consists not only of GC-EI-MS data used for the QSAR modeling but also includes the rest of the GC-EI-MS data (n = 49007).

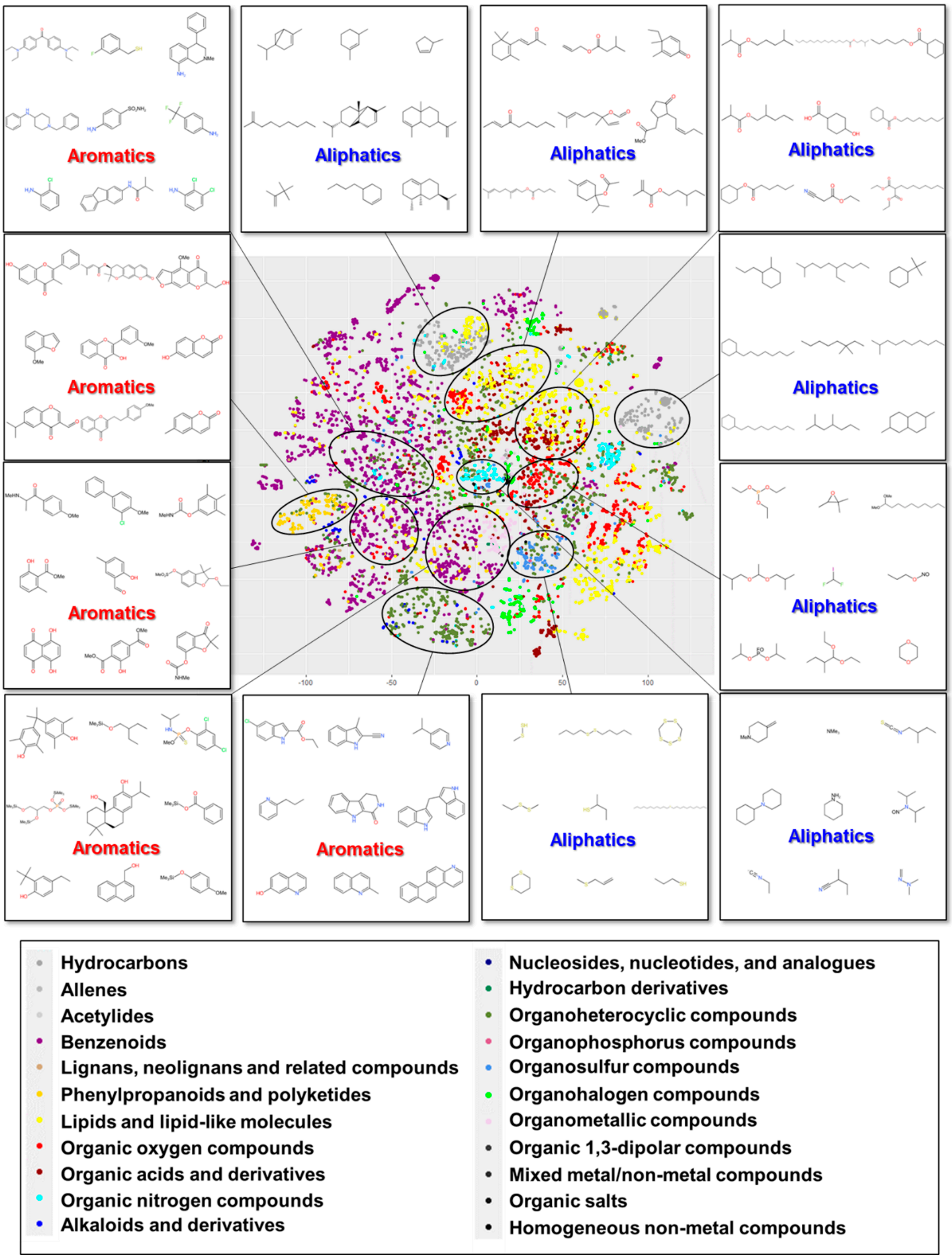

Figure S4. t-SNE map based on a molecular descriptor (PubChem fingerprint)

The plot consists of dataset of the descriptor used for the QSAR modeling (n = 12785).

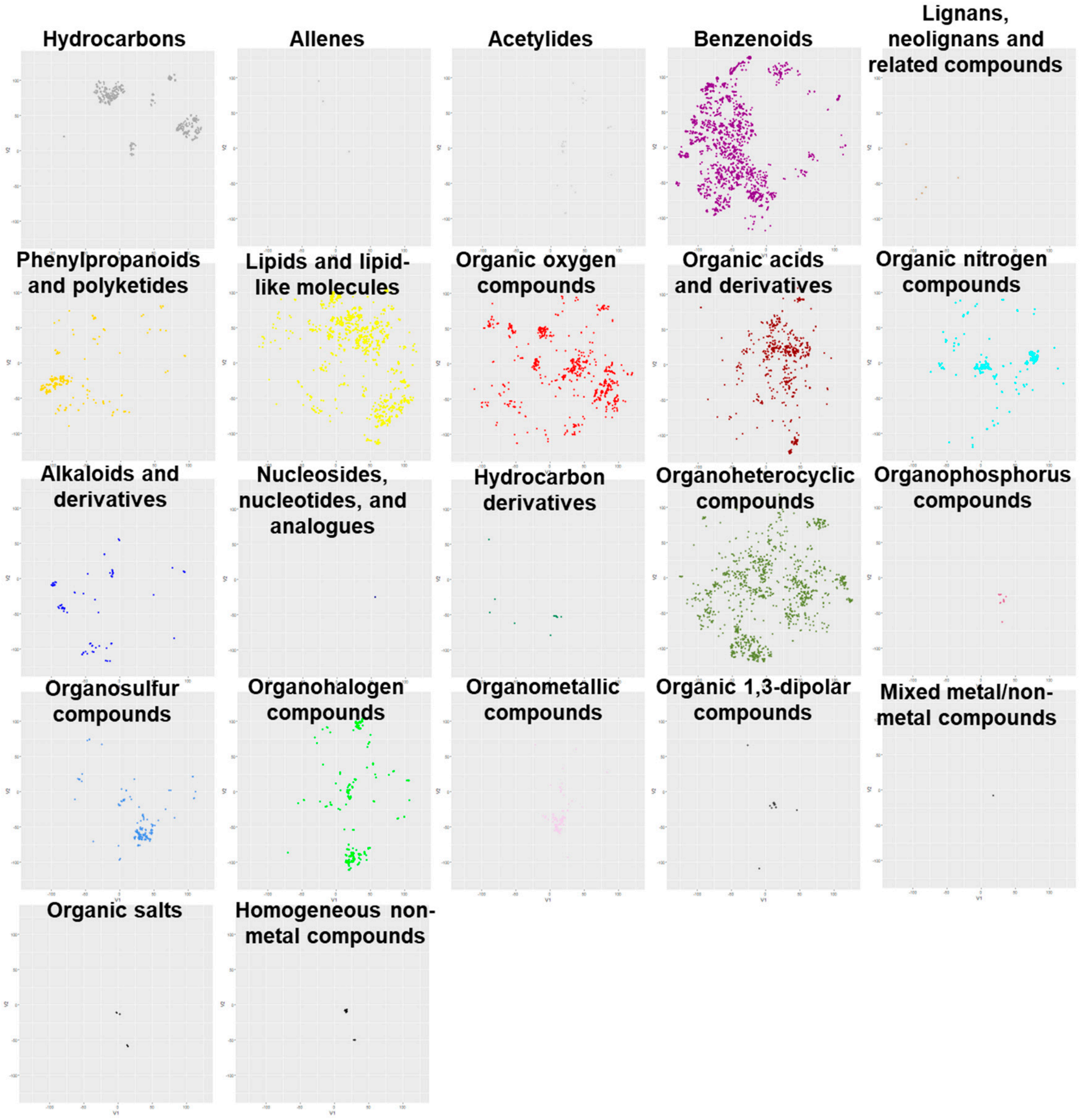

Figure S5. t-SNE map based on molecular descriptor (PubChem fingerprint) for each superclass by ClassyFire

The colors of the plots indicate the superclasses of chemicals obtained using ClassyFire. The circled clusters were designated as aliphatics, aromatics, or mixed, based on the chemical structures of their predominant members. See Figure S-4 for the color legend.

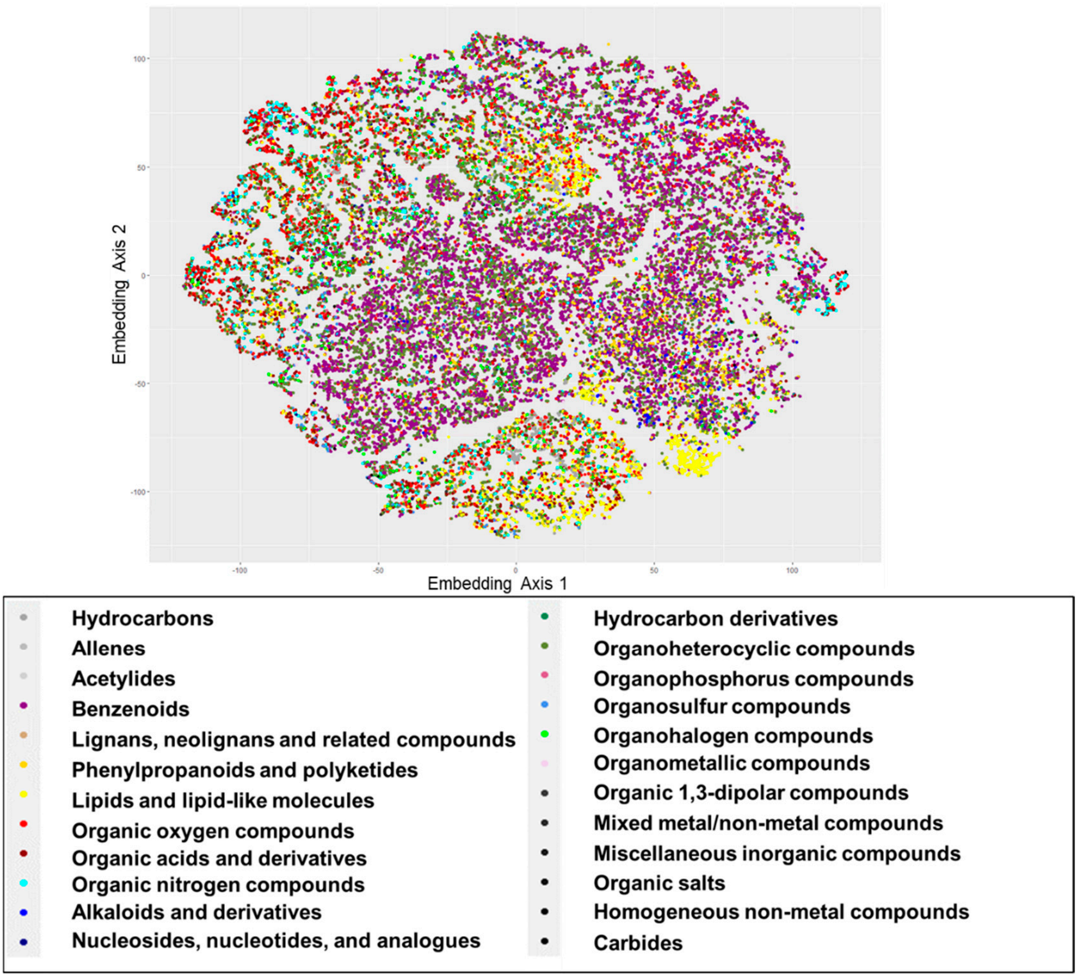

Figure S6. t-SNE map based on standardized analytical descriptor

The plot consists not only of GC-EI-MS data used for the QSAR modeling but also includes the rest of the GC-EI-MS data (*n* = 49007).

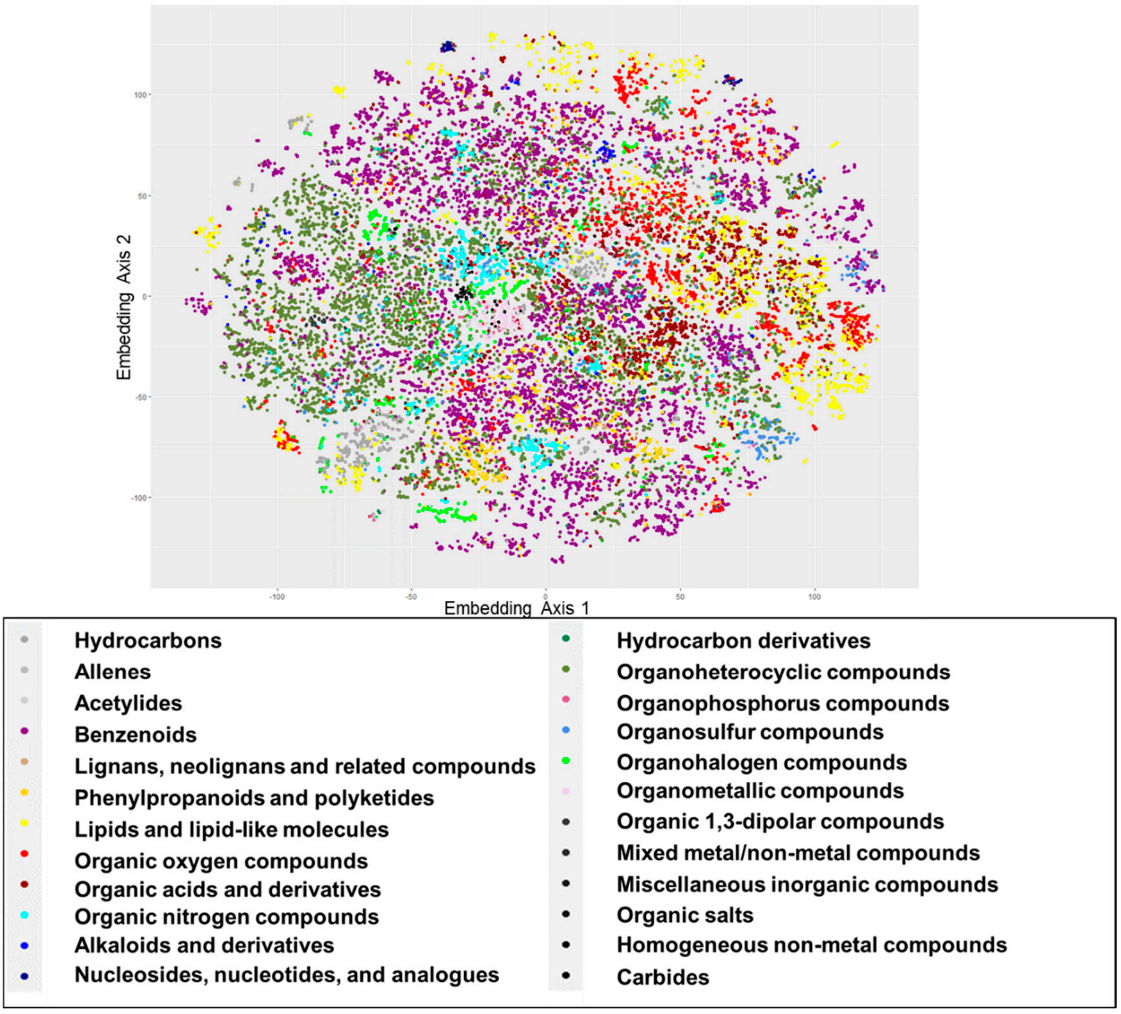

Figure S7. t-SNE map based on molecular descriptor (PubChem fingerprint)

The plot consists not only of dataset of the descriptor used for the QSAR modeling but also includes the rest of the data (n = 49007).

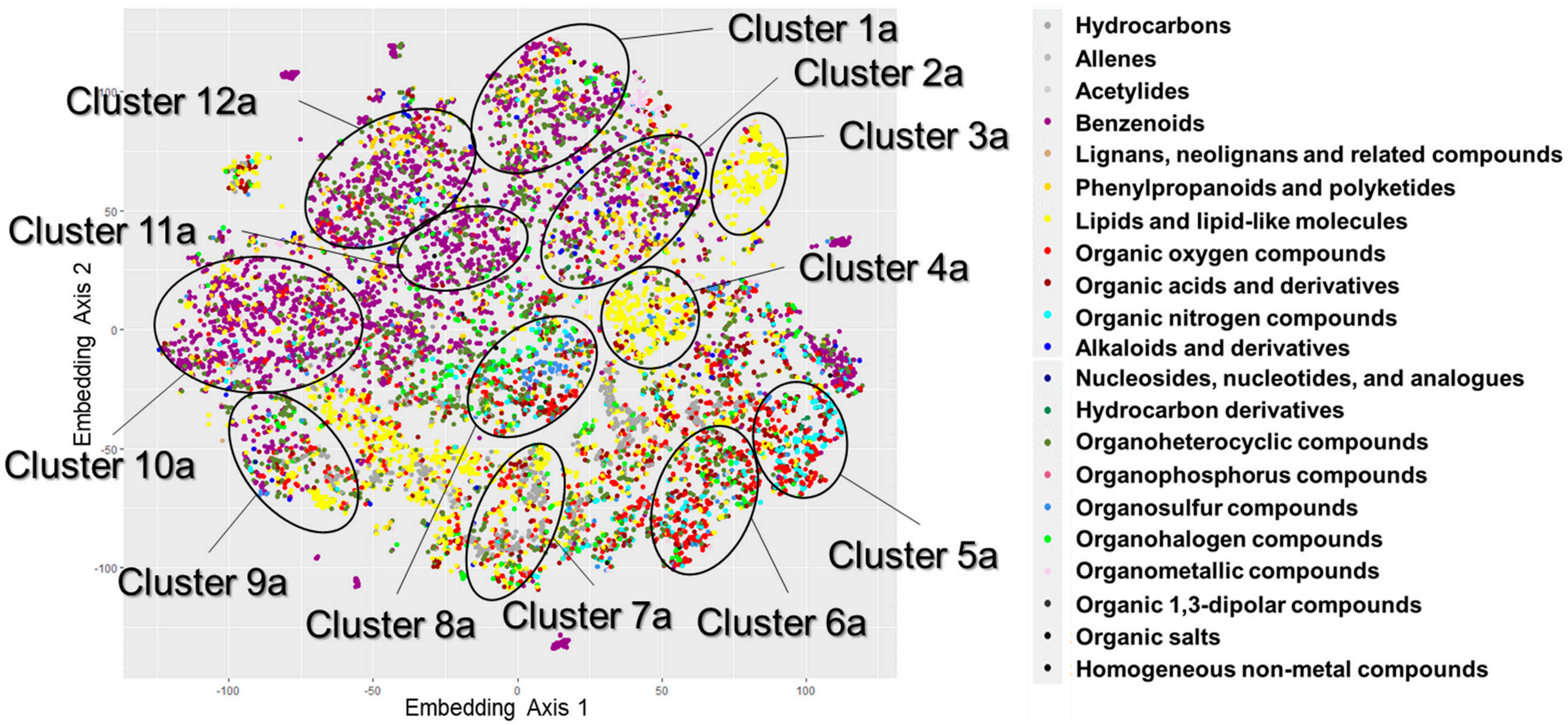

Figure S8. Cluster IDs of analytical-descriptor-based t-SNE map used for calculating cosine similarities The plot consists of GC-EI-MS data with RI that are available for the QSAR modeling (n = 12859).

Table S1. Cosine similarities of standardized mass spectra between/within analytical-descriptor-based t-SNE clusters

	Cluster 1a	Cluster 2a	Cluster 3a	Cluster 4a	Cluster 5a	Cluster 6a	Cluster 7a	Cluster 8a	Cluster 9a	Cluster 10a	Cluster 11a	Cluster 12a
Cluster 1a	0.086 ± 0.159											
Cluster 2a	0.001 ± 0.040	0.027 ± 0.091										
Cluster 3a	0.029 ± 0.094	0.005 ± 0.065	0.234 ± 0.176									
Cluster 4a	–0.015 ± 0.073	–0.007 ± 0.047	–0.018 ± 0.052	0.137 ± 0.150								
Cluster 5a	–0.003 ± 0.052	–0.008 ± 0.045	–0.103 ± 0.061	0.025 ± 0.091	0.219 ± 0.219							
Cluster 6a	–0.020 ± 0.047	–0.018 ± 0.037	–0.084 ± 0.054	0.059 ± 0.103	0.163 ± 0.168	0.198 ± 0.183						
Cluster 7a	–0.055 ± 0.042	–0.028 ± 0.035	–0.018 ± 0.060	0.115 ± 0.124	0.023 ± 0.081	0.102 ± 0.133	0.319 ± 0.219					
Cluster 8a	–0.015 ± 0.053	–0.018 ± 0.027	–0.044 ± 0.040	0.040 ± 0.099	0.054 ± 0.109	0.099 ± 0.128	0.039 ± 0.103	0.135 ± 0.156				
Cluster 9a	–0.022 ± 0.042	–0.016 ± 0.030	0.018 ± 0.084	–0.016 ± 0.051	0.021 ± 0.061	0.020 ± 0.069	0.057 ± 0.111	–0.009 ± 0.056	0.120 ± 0.190			
Cluster 10a	0.023 ± 0.078	–0.008 ± 0.035	0.021 ± 0.093	–0.055 ± 0.039	0.031 ± 0.051	0.001 ± 0.046	–0.050 ± 0.058	–0.030 ± 0.041	0.022 ± 0.083	0.120 ± 0.179		
Cluster 11a	0.008 ± 0.065	–0.002 ± 0.040	–0.036 ± 0.046	–0.009 ± 0.069	–0.009 ± 0.039	–0.024 ± 0.038	–0.040 ± 0.058	–0.013 ± 0.050	–0.019 ± 0.046	0.001 ± 0.060	0.071 ± 0.154	
Cluster 12a	–0.008 ± 0.030	0.004 ± 0.043	–0.035 ± 0.041	–0.024 ± 0.038	0.005 ± 0.042	–0.014 ± 0.034	–0.033 ± 0.044	–0.023 ± 0.030	–0.003 ± 0.051	0.010 ± 0.052	0.013 ± 0.076	0.049 ± 0.127

The values represent the median ± standard deviation.

See Figure S8 for each cluster ID.

Table S2. Cosine similarities of standardized mass spectra between/within molecular-descriptor-based t-SNE clusters

	Cluster 1b	Cluster 2b	Cluster 3b	Cluster 4b	Cluster 5b	Cluster 6b	Cluster 7b	Cluster 8b	Cluster 9b	Cluster 10b	Cluster 11b	Cluster 12b
Cluster 1b	0.338 ± 0.234											
Cluster 2b	0.292 ± 0.193	0.301 ± 0.193										
Cluster 3b	0.207 ± 0.181	0.242 ± 0.176	0.334 ± 0.206									
Cluster 4b	0.288 ± 0.222	0.300 ± 0.187	0.342 ± 0.223	0.434 ± 0.286								
Cluster 5b	0.162 ± 0.166	0.178 ± 0.158	0.233 ± 0.188	0.233 ± 0.208	0.203 ± 0.195							
Cluster 6b	0.138 ± 0.154	0.143 ± 0.146	0.174 ± 0.160	0.180 ± 0.174	0.144 ± 0.157	0.152 ± 0.169						
Cluster 7b	0.185 ± 0.166	0.188 ± 0.158	0.230 ± 0.186	0.229 ± 0.205	0.193 ± 0.167	0.140 ± 0.147	0.244 ± 0.184					
Cluster 8b	0.078 ± 0.090	0.082 ± 0.093	0.063 ± 0.078	0.056 ± 0.074	0.058 ± 0.089	0.042 ± 0.071	0.064 ± 0.074	0.084 ± 0.136				
Cluster 9b	0.066 ± 0.090	0.061 ± 0.079	0.041 ± 0.054	0.041 ± 0.060	0.035 ± 0.053	0.038 ± 0.069	0.045 ± 0.057	0.051 ± 0.084	0.062 ± 0.116			
Cluster 10b	0.086 ± 0.093	0.087 ± 0.096	0.055 ± 0.075	0.062 ± 0.086	0.044 ± 0.063	0.038 ± 0.067	0.054 ± 0.067	0.074 ± 0.113	0.055 ± 0.084	0.106 ± 0.156		
Cluster 11b	0.067 ± 0.079	0.067 ± 0.077	0.046 ± 0.069	0.047 ± 0.076	0.034 ± 0.055	0.030 ± 0.051	0.044 ± 0.059	0.058 ± 0.079	0.050 ± 0.068	0.072 ± 0.103	0.072 ± 0.101	
Cluster 12b	0.077 ± 0.089	0.077 ± 0.088	0.062 ± 0.089	0.065 ± 0.096	0.051 ± 0.076	0.046 ± 0.076	0.058 ± 0.079	0.058 ± 0.082	0.055 ± 0.089	0.068 ± 0.094	0.060 ± 0.078	0.068 ± 0.104

The values represent the median ± standard deviation. The molecular descriptor was the PubChem fingerprint.

See Figure S8 for each cluster ID.

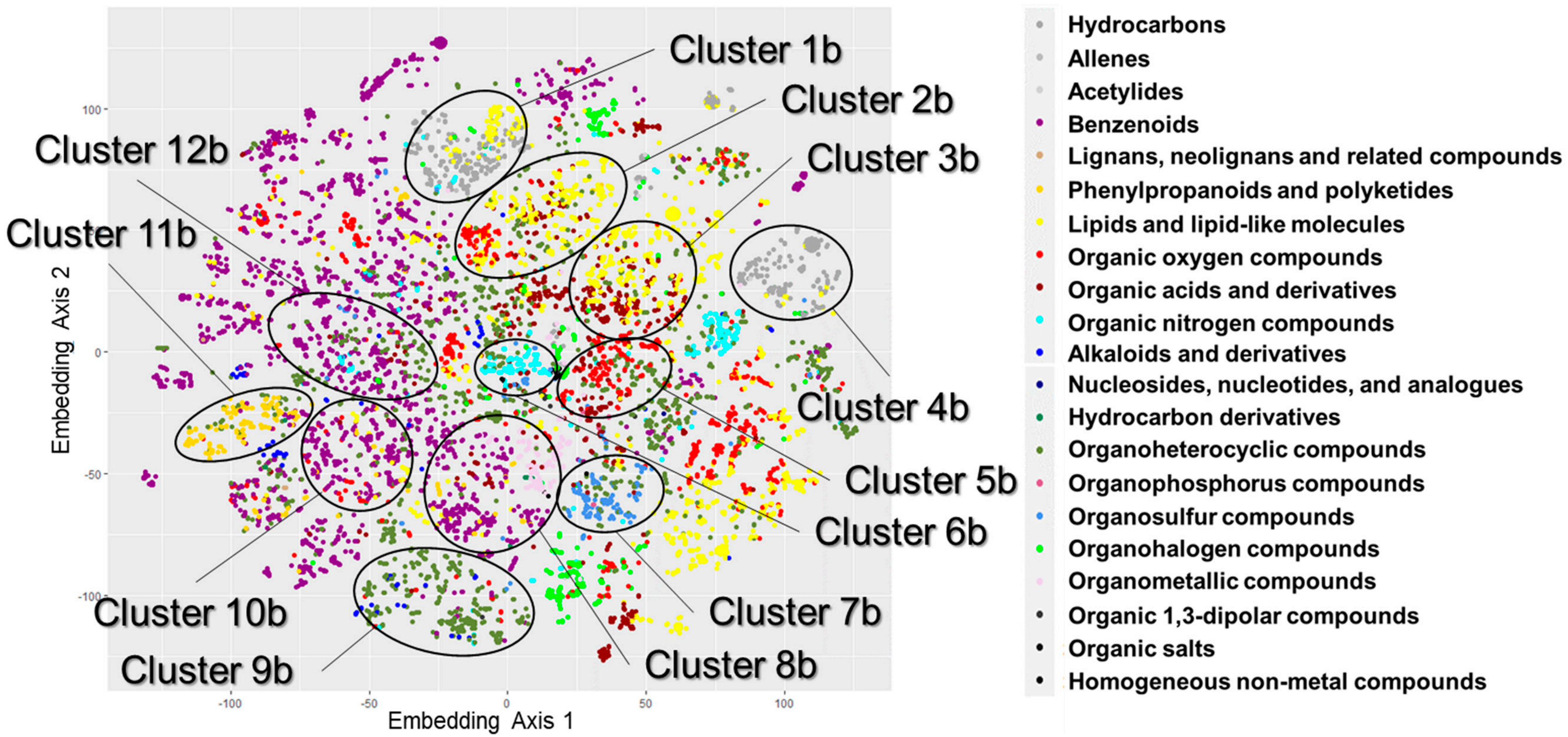

Figure S9. Cluster IDs of molecular-descriptor-based t-SNE map used for calculating cosine similarities

The molecular descriptor was the PubChem fingerprint (n = 12785).

Table S3. Cosine similarities of randomly selected 200 standardized mass spectra inter/intra-superclass by ClassyFire

	Organic oxygen compounds	Organic acids and derivatives	Benzenoids	Hydrocarbons	Organoheterocyclic compounds	Lipids and lipid-like molecules
Organic oxygen compounds	0.060 ± 0.156 (*n* = 19,900)					
Organic acids and derivatives	0.053 ± 0.141 (*n* = 40,000)	0.063 ± 0.153 (*n* = 19,900)				
Benzenoids	–0.017 ± 0.067 (*n* = 40,000)	–0.014 ± 0.063 (*n* = 40,000)	0.019 ± 0.088 (*n* = 19,900)			
Hydrocarbons	0.068 ± 0.160 (*n* = 40,000)	0.061 ± 0.163 (*n* = 40,000)	–0.033 ± 0.067 (*n* = 40,000)	0.174 ± 0.261 (*n* = 19,900)		
Organoheterocyclic compounds	0.011 ± 0.097 (*n* = 40,000)	0.008 ± 0.086 (*n* = 40,000)	0.0001 ± 0.066 (*n* = 40,000)	0.005 ± 0.106 (*n* = 40,000)	0.009 ± 0.085 (*n* = 19,900)	
Lipids and lipid-like molecules	0.024 ± 0.131 (*n* = 40,000)	0.017 ± 0.128 (*n* = 40,000)	–0.021 ± 0.071 (*n* = 40,000)	0.063 ± 0.169 (*n* = 40,000)	–0.005 ± 0.089 (*n* = 40,000)	0.054 ± 0.155 (*n* = 19,900)

The values represent the median ± standard deviation.

Table S4. MAE-based predictive performances of analytical- and molecular-descriptor-based QSAR models

	Detective-QSAR	Topological Descriptor	ECFP6	MACCS Key	PubChem Fingerprint
log Mw	0.027	0.003	0.034	0.038	0.026
log $K_{o\text{-}w}$	0.762	0.356	0.596	0.541	0.408
BP	13.6	11.5	30.1	25.2	17.9
MP	39.2	29.6	37.7	37.0	32.2
log WS	1.01	0.54	0.92	0.77	0.57
log LD_{50} (rat, oral)	0.53	0.44	0.48	0.44	0.42
log LD_{50} (mouse, oral)	0.52	0.41	0.42	0.41	0.40

Table S5. Number of training data needed for analytical- and molecular-descriptor-based QSAR models.

	Detective-QSAR	Topological Descriptor	ECFP6	MACCS Key	PubChem Fingerprint
log Mw	10248	10224	10224	10224	10224
log $K_{o\text{-}w}$	2140	2120	2120	2120	2120
BP	2709	2603	2603	2603	2603
MP	3068	2983	2983	2983	2983
log WS	1107	1100	1100	1100	1100
log LD_{50} (rat, oral)	1664	1643	1643	1643	1643
log LD_{50} (mouse, oral)	1304	1290	1290	1290	1290

Table S6. MAE-based predictive performances of various models

	Detective-QSAR	Detective-QSAR (without RI)	All-six-variable model	maxint_mz	RI	centroid_mz
log Mw	0.027	0.101	0.040	0.040	0.072	0.106
log $K_{o\text{-}w}$	0.76	0.68	1.08	1.17	1.37	1.16
BP	13.6	29.2	19.9	45.8	20.3	49.4
MP	39.2	43.8	48.0	64.0	52.0	60.8
log WS	1.01	0.93	1.30	1.45	1.65	1.37
log LD_{50} (rat, oral)	0.53	0.49	0.60	0.60	0.61	0.63
log LD_{50} (mouse, oral)	0.52	0.48	0.53	0.53	0.55	0.56

QSAR model was based on analytical descriptors; regression models were based on indicators of GC-EI-MS data. All-six-variable model: centroid_mz + maxint_mz + max_mz + sd_mz + bin_num + RI.

Table S7. Number of training data used for various models

	Detective-QSAR	Detective-QSAR (without RI)	All-six-variable model	maxint_mz	RI	centroid_mz
log Mw	10248	10236	10236	10236	10236	10236
log $K_{o\text{-}w}$	2140	2140	2119	2119	2119	2119
BP	2709	2709	2615	2615	2615	2615
MP	3068	3068	3047	3047	3047	3047
log WS	1107	1107	1101	1101	1101	1101
log LD_{50} (rat, oral)	1664	1643	1810	1810	1810	1810
log LD_{50} (mouse, oral)	1304	1290	1290	1290	1290	1290

QSAR model was based on analytical descriptors; regression models were based on indicators of GC-EI-MS data. All-six-variable model: centroid_mz + maxint_mz + max_mz + sd_mz + bin_num + RI.
